# The gut microbiota and post-traumatic major depression disorder: insights from bidirectional two-sample Mendelian randomization

**DOI:** 10.3389/fpsyt.2024.1383664

**Published:** 2024-05-14

**Authors:** Shan Liu, Yu Wang, Yonghu Zhang, Liang Zeng, Lin Ling, Yong Luo, Wenjie Liu

**Affiliations:** ^1^ The Second Affiliated Hospital, Department of Emergency, Hengyang Medical School, University of South China, Hengyang, Hunan, China; ^2^ The Second Affiliated Hospital, Department of Anesthesiology, Hengyang Medical School, University of South China, Hengyang, Hunan, China

**Keywords:** post-traumatic major disorder, gut microbiota, microbiota-gut-brain axis, Mendelian randomization, bidirectional

## Abstract

**Background:**

Exposure to trauma is often associated with an increased incidence of Major Depressive Disorder (MDD), yet the mechanisms underlying MDD development post-trauma remain elusive. The microbiota-gut-brain axis has been implicated in neuropsychiatric disorders, suggesting its potential role in post-traumatic MDD (PTMDD) development. Our study aimed to assess the significance of the gut microbiome-brain interaction in PTMDD.

**Methods:**

We conducted a bidirectional two-sample Mendelian Randomization (MR) analysis to investigate the causal relationship between the gut microbiota and both PTMDD and trauma exposure in MDD. Genome-wide association study (GWAS) summary datasets for PTMDD and trauma exposure in MDD, both derived from the UK Biobank. The PTMDD dataset included 24,090 individuals (13,393 cases and 10,701 controls), while the dataset for trauma exposure in MDD comprised 22,880 participants (13,393 cases and 9,487 controls). Additionally, gut microbiota data from the MiBioGen consortium included 14,306 European individuals across 18 diverse cohorts.

**Results:**

Our research identified a significant negative association between the phylum Verrucomicrobia (odds ratio (OR) [95% confidence interval (CI)] =0.799 [0.684–0.933], P=0.005) and the risk of developing PTMDD, suggesting a protective role for Verrucomicrobia against PTMDD. Conversely, our findings indicate no causal effects of the gut microbiota on trauma exposure in MDD. However, reverse analysis revealed that both PTMDD and MDD influence certain bacterial traits, affecting 5 and 9 bacterial traits, respectively. Moreover, Verrucomicrobia (OR [95% CI] = 1.166 [1.051 - 1.294], P=0.004) was found to be positively impacted by trauma exposure in MDD.

**Conclusion:**

Our findings provide a cause-and-effect relationship between the gut microbiota and PTMDD, contributing to our understanding of the microbiota-gut-brain axis and its role in neuropsychiatric disorder development after trauma. This information provides an opportunity for new treatment and prevention methods which are aimed at the gut-brain interaction.

## 1 Introduction

Major Depressive Disorder (MDD) stands as one of the major global health issues, with approximately 350 million individuals suffering from it around the world (1). It contributes 5.5% of the total disability-adjusted life years, significantly affecting individuals and societies (2). The likelihood of developing MDD is even more common regarding traumatic experiences, especially those that arise in early life (3, 4). The brain chemistry and the stress response system can be altered by exposure to severe or chronic trauma and this can result in a range of mental health disorders, including MDD (5, 6). Though the relationship between MDD and a trauma exposure is acknowledged, the mechanisms implicated in the development of post-traumatic MDD (PTMDD) are not yet clear. This knowledge gap hampers the efficacy of some preventive and therapeutic approaches (7, 8). Therefore, more studies need to be conducted to reveal the pathophysiology of PTMDD.

The microbiome-gut-brain axis, a neural, hormonal, and immunological signaling system, has been extensively described in recent studies (9–11). However, this interplay is critically influenced by the gut microbiota, whose metabolites and neurochemicals affect brain function and behavior (8, 12). A traumatic insult can alter the structure and richness of the gut microbiota, thereby affecting the microbiome-gut-brain axis (8, 13). These alterations may manifest as traumatic psychiatric sequelae (13, 14).These findings suggest that the microbiome-gut-brain axis may play a vital role in the development of post-traumatic neuropsychiatric sequelae.

Malan-Muller et al.’s research linked the gut microbiota and post-traumatic stress disorder (PTSD). In the stool samples, they identified and separated specific patterns of microorganisms that were associated with PTSD, and four bacteria were found to be potentially indicative of this condition (14). In addition, Abigail L. Zeamer et al. pointed out that microbiomes are involved in the development of post-traumatic neuropsychiatric sequelae, indicating that such microbiomes can modulate brain function through global arginine bioavailability reduction (8). Dennis W. Simon et al., stated that depletion of the microbiome might enhance neurological results in people with traumatic brain injury (15). Research conducted in animals indicates that trauma may change the microbiome, which in turn causes the increase of inflammation both locally and systemically (16). On the whole, these research work hint that the microbiome-gut-brain axis may be a key to figuring out and treating trauma-related disorders such as PTMDD.

While current evidence, primarily from associative studies, suggests a link between gut microbiota alterations and the onset of PTMDD, establishing a definitive causal relationship remains challenging. To progress from mere correlation to causation, employing more robust research methods such as randomized controlled trials (RCTs) and Mendelian Randomization (MR) is crucial. These methods are poised to offer more definitive evidence, elucidating causal mechanisms and potentially revolutionizing therapeutic and preventative strategies for PTMDD (17). Compared to animal models and observational studies, RCTs could provide more reliable evidence. However, conducting RCTs in this area faces several challenges, including ethical considerations related to manipulating the gut microbiota, the complexity of maintaining blinding during interventions, technical limitations in characterizing the gut microbiota, the necessity for large samples, and prolonged follow-up periods to observe meaningful clinical outcomes, as well as the confounding effects of comorbid conditions and concurrent treatments in participants with PTMDD. Each of these factors presents significant difficulties.MR employs genetic variants as proxies to investigate causality in PTMDD, thereby overcoming the challenges associated with direct interventions typical of RCTs. For instance, this method utilizes naturally occurring genetic variations, effectively eliminating the need for experimental manipulation, which can raise ethical concerns, particularly when modifying health-related behaviors or biological systems. Consequently, MR emerges as an alternative choice.

In this study, we employed a bidirectional two-sample MR analysis to investigate the causal relationships between gut microbiota and both PTMDD and trauma exposure in MDD, which shared the same cases. The distinct controls for each condition were chosen to provide more robust evidence and to identify the distinctions between those specifically diagnosed with PTMDD and the effects of trauma in the general MDD population. Our study aims to provide a deeper understanding of the role of the microbiome-gut-brain axis in PTMDD and to develop novel interventions by targeting specific profiles in its pathogenesis.

## 2 Materials and methods

### 2.1 Ethics statement

No additional ethical approvals were required because all of the genome-wide association study (GWAS) summary datasets used in our study have been previously published.

### 2.2 Study design

**Figure 1:**
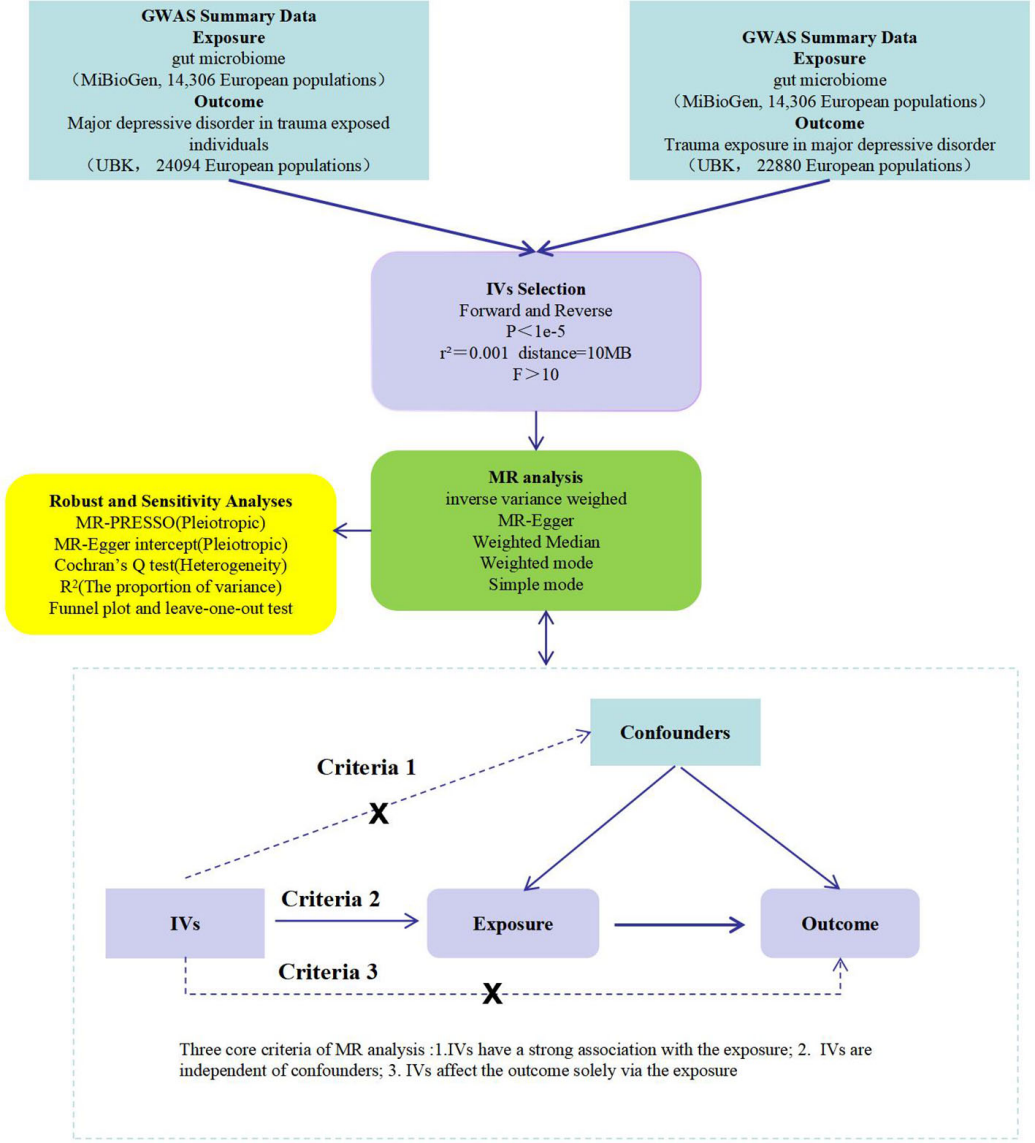
A flow chart of this study. GWAS, genome-wide association study; IVs, instrumental variables; MR, mendelian randomization; MR-PRESSO, MR pletropy residual sum and outlier; PTMDD, post-traumatic major depression disorder.

This study first examines the influence of gut microbiota on PTMDD and trauma exposure in MDD using a forward MR analysis. In the reverse MR analysis, we then assess the potential causal effects of PTMDD and trauma exposure in MDD on the composition of gut microbiota. These bidirectional analyses are conducted using a two-sample MR approach, utilizing GWAS summary data to provide more robust evidence and to identify distinctions between these conditions.The design adheres to MR analysis’s three criteria: (1)Instrumental variables (IVs) have a strong association with the exposure; (2) IVs are independent of confounders; and (3) IVs affect the outcome solely via the exposure (18).This is detailed in **Figure 1**.

### 2.3 Data sources

Our study utilized gut microbiota data sourced from the MiBioGen consortium, an entity that compiles and analyzes genome-wide genotypes alongside 16S fecal microbiome data (19). More specifically, our analysis was focused on the European sub-set of the consortium’s data, which comprises 14,306 individuals within 18 separate cohorts. This targeted approach was chosen in line with our research goals and for the sake of demographic consistency within our sample population. Throughout the data curation process, we deliberately excluded any unidentified bacterial strains, a measure taken to enhance the precision and relevance of our dataset (https://mibiogen.gcc.rug.nl/).

The data utilized in our study for PTMDD and trauma exposure in MDD were derived from GWAS data, as detailed in the specific publication (4). These data were obtained from the UK Biobank, which includes health-related phenotypes and genome-wide genotype information from approximately 500,000 British individuals aged 40 to 70 years. Our study’s subset of data consists of responses from 157,366 participants who completed an online follow-up questionnaire. This questionnaire assessed mental health disorders, including symptoms of MDD, and contained 16 items regarding traumatic events. Phenotypic data for MDD were extracted from this questionnaire, utilizing definitions from recent publications. The identification of probable MDD cases was based on responses aligned with the Composite International Diagnostic Interview (CIDI), excluding those with self-reported schizophrenia, other psychoses, or bipolar disorder. Individuals reporting mental illnesses or indicators of mood disorders were excluded from the control group. Traumatic experiences were assessed using both the Childhood Trauma Screener and an analogous screener for adult experiences. The inclusion criteria for trauma exposure were strictly based on the correlation of specific reported events with MDD. Following the application of these criteria, our final sample size of PTMDD for detailed analysis was refined to 24,094 participants (13,393 cases and 10,701 controls) (https://gwas.mrcieu.ac.uk/datasets/ebi-a-GCST009980/), Additionally, the final sample size for examining trauma exposure in MDD was 22,880 participants (13,393 cases and 9,487 controls) (https://gwas.mrcieu.ac.uk/datasets/ebi-a-GCST009983/) (**Supplementary Table 1**).

### 2.4 Instrumental variable selection

Due to the distinctive features of bacterial taxa, we divided them into five categories for analysis: phylum, class, order, family, and genus. Initially, IVs were filtered assuming p < 1 × 10–5 criterion for providing more complete outcomes. Afterwards, these IVs were subjected to linkage disequilibrium (LD) clumping with parameters defined as r^2 =^ 0.001 with a distance threshold of 10Mb to address the issue of single nucleotide polymorphisms (SNPs) correlations. SNPs with allele inconsistencies across the exposure and outcome datasets and palindromic SNPs with intermediate allele frequencies were also eliminated from the analysis. Finally, In order to access the strength of our genetic instruments for exposures, we calculated the R² and F statistic. These calculation used the formula: R2 = 2 × b2 × EAF × (1 − EAF)/(2 × b2 × EAF × (1− EAF) + 2 × SE2 × N × EAF × (1 − EAF)), and F = (N - k - 1)/k × (R²/(1 - R²)), where R² is the cumulative variance explained by the chosen SNPs, N is the sample size, and k is the count of SNPs utilized in our study. An F statistic exceeding 10 is indicative of sufficient instrument strength, thereby minimizing the risk of weak instrument bias in our two-sample model (20).

### 2.5 Statistical analysis

After selecting proper IVs, we conducted five MR methods—inverse-variance weighted (IVW), MR-Egger, simple mode, weighted mode, and weighted median—to investigate the causal relationship between the exposure and the outcome. Our primary results were derived from the IVW method and further supported by the other four methodologies (21).Cochran’s Q statistic in the IVW meta-analysis was employed to assess statistical heterogeneity (22, 23). To evaluate pleiotropy, we utilized the MR-Egger intercept and MR-PRESSO approaches (22). Moreover, a leave-one-out sensitivity test and funnel plot were conducted to ensure the robustness and consistency of our causal effect estimates (24).Additionally, the MR Steiger method was employed to ascertain whether the exposure had a directional causal effect on the outcome.

All tests were two-sided, and the p-value threshold was set based on the Bonferroni correction method (p < 0.05/n). We established the significance thresholds for each bacterial taxa as follows: for the phylum, p = 5.56 × 10^−3^ (0.05/9), for the class p = 3.13 × 10^−3^ (0.05/16), for the order p = 2.5 × 10^−3^ (0.05/20), for the family p = 1.43 × 10^−3^ (0.05/35), and for the genus p= 3.82 × 10^−4^ (0.05/131). Because the reverse MR analysis examines the effects of both PTMDD and trauma exposure in MDD on various gut microbiota, we have set the significance threshold at p = 0.025 (0.05/2). All statistical analyses were performed using R software (version 4.3.2) and the R packages “TwoSampleMR,” “fastMR,” and “MRPRESSO”.

## 3 Results

### 3.1 Instrumental variables selection

We screened 2,362 SNPs to be used as instrumental variables (IVs), which included 211 bacterial traits. The F-statistics for these SNPs all exceeded the threshold of 10, indicating strong instrument strength (**Supplementary Table 2**). The instrumental variables (IVs) detailed in **Supplementary Tables 3**, **4**.

### 3.2 The causal relationship between gut microbiota and PTMDD

**Figure 2:**
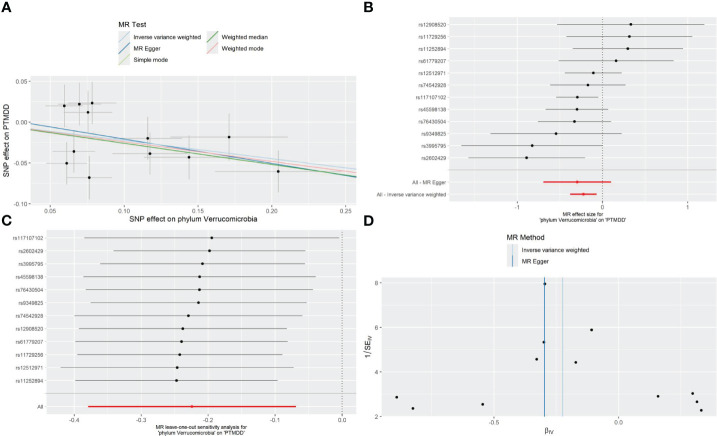
Forest plot **(A)**, sensitivity analysis **(B)**, scatter plot **(C)**, and funnel plot **(D)** of the causal effect of the phylum Verrucomicrobia on the risk of development of PTMDD.

As detailed in **Table 1**, the phylum Verrucomicrobia (odds ratio (OR) [95% confidence interval (CI)] =0.799 [0.684–0.933], P=0.005), reached a Bonferroni-corrected significance level in the IVW analysis. Further analyses, including MR Egger, Weighted Median, Simple Mode, and Weighted Mode, are detailed in **Table 1**.We assessed the IVs’ horizontal pleiotropy and heterogeneity using MR-Egger regression and Cochran’s Q statistic, finding neither in the relationship between the IVs and the outcome. Further, our gut microbiota analysis showed no outliers, and MR-PRESSO results confirmed no significant deviations. We used funnel plot and leave-one-out sensitivity analysis to further validate our results, confirming the reliability and stability of our causal effect estimates (**Figure 2**).

**Table 1:** MR analysis of the gut microbiota on PTMDD.

Classification	Methods	Nsnp	Beta	SE	OR(95%CI)	P value	Horizontal pleiotropy			Heterogeneity		MR-PRESSO	
							Egger intercept	SE	P value	Cochran’s Q	P value	outlier	P value
Phylum Verrucomicrobia	IVW	12	-0.224	0.079	0.799(0.684–0.933)	0.005	0.009	0.023	0.7	14.691	0.144	0	0.285
	MR Egger		-0.297	0.202	0.743(0.5–1.104)	0.172							
	Weighted median		-0.259	0.096	0.772(0.639–0.932)	0.007							
	Simple mode		-0.241	0.147	0.786(0.589–1.048)	0.129							
	Weighted mode		-0.241	0.099	0.786(0.647–0.955)	0.033							

MR, Mendelian randomization; PTMDD, Post-traumatic major depression disorder; Nsnp, Numbers of single nucleotide polymorphism; SE, Standard error; OR, Odds ratio; CI, Confidence interval; IVW, Inverse variance weighted.

In the reverse MR analysis, significant results from the IVW analysis indicated that PTMDD was positively correlated with specific bacterial traits, including the genus Anaerotruncus (OR [95% CI] = 1.384 [1.118**–**1.715], P = 0.003), genus Butyrivibrio (OR [95% CI]= 1.164 [1.023**–**1.325], P = 0.021), phylum Lentisphaerae (OR [95% CI] = 1.185 [1.022**–**1.374], P = 0.024), and family Rhodospirillaceae (OR [95% CI] = 1.178 [1.018**–**1.364]; P = 0.028). In contrast, PTMDD was negatively correlated with the family Bacteroidales S24 7 group (OR [95% CI]=[0.791**–**0.964], P = 0.007) and genus Howardella (OR [95% CI] = 0.878 [0.773**–**0.998], P = 0.047) (**Table 2**).

**Table 2:** MR analysis of PTMDD on the gut microbiota.

Classification	Methods	Nsnp	Beta	SE	OR(95%CI)	P value	Horizontal pleiotropy			Heterogeneity		MR-PRESSO	
							Egger intercept	SE	P value	Cochran’s Q	P value	outlier	P value
family Bacteroidales S24 7group	IVW	11	-0.136	0.05	0.873 (0.791- 0.964)	0.007	0.13	0.328	0.724	12.405	0.191	0	0.321
	MR Egger		-1.241	3.035	0.289 (0.001- 110.860)	0.692							
	Weighted median		-0.129	0.065	0.879 (0.774- 0.999)	0.047							
	Simple mode		-0.187	0.099	0.830 (0.684- 1.007)	0.087							
	Weighted mode		-0.168	0.105	0.845 (0.688, 1.038)	0.14							
class Bacilli	IVW	11	0.071	0.03	1.074 (1.011- 1.139)	0.02	-0.001	0.205	0.999	5.484	0.79	0	0.868
	MR Egger		0.057	0.042	1.059 (0.976- 1.149)	0.968							
	Weighted median		0.045	0.06	1.046 (0.931- 1.177)	0.171							
	Simple mode		0.043	0.055	1.044 (0.937- 1.164)	0.465							
	Weighted mode		0.043	0.055	1.044 (0.937–1.164)	0.453							
genus Butyricimonas	IVW	11	0.084	0.039	1.088 (1.009- 1.174)	0.029	0.415	0.261	0.147	6.828	0.655	0	0.536
	MR Egger		-3.44	2.223	0.032 (0.000- 2.504)	0.156							
	Weighted median		0.141	0.054	1.151 (1.035–1.280)	0.01							
	Simple mode		0.162	0.097	1.176 (0.971–1.423)	0.127							
	Weighted mode		0.162	0.092	1.176 (0.981–1.409)	0.11							
order Lactobacillales	IVW	11	0.066	0.03	1.068 (1.006–1.134)	0.03	-0.010	0.206	0.963	4.232	0.775	0	0.895
	MR Egger		0.15	1.748	1.162 (0.038, 35.722)	0.934							
	Weighted median		0.057	0.038	1.059 (0.983- 1.141)	0.132							
	Simple mode		0.047	0.062	1.048 (0.929–1.182)	0.465							
	Weighted mode		0.044	0.054	1.045 (0.941–1.161)	0.431							
genus Howardella	IVW	11	-0.13	0.065	0.878 (0.773- 0.998)	0.047	0.614	0.45	0.209	2.658	0.954	0	0.894
	MR Egger		-5.346	3.822	0.005 (0.000–8.547)	0.199							
	Weighted median		-0.125	0.085	0.882 (0.746–1.043)	0.142							
	Simple mode		-0.005	0.138	0.995 (0.759- 1.305)	0.972							
	Weighted mode		-0.009	0.146	0.991 (0.745- 1.319)	0.955							

MR, Mendelian randomization; PTMDD, Post-traumatic major depression disorder; Nsnp, Numbers of single nucleotide polymorphism; SE, Standard error; OR, Odds ratio; CI, Confidence interval; IVW, Inverse variance weighted.

### 3.3 The causal relationship between gut microbiota and trauma exposure in MDD

In the forward MR analysis, we found that no result from the IVW analysis reached a Bonferroni-corrected significance level.

In the reverse MR analysis, detailed in **Table 3**, we observed positive correlations with the family Verrucomicrobiaceae (OR [95% CI] = 1.191 [1.066 - 1.33], P=0.002), order Verrucomicrobiales (OR [95% CI] = 1.19 [1.065 - 1.329], P=0.002), class Verrucomicrobiae (OR [95% CI] = 1.19 [1.065 - 1.329], P=0.002), genus Akkermansia (OR [95% CI] = 1.19 [1.065 - 1.33], P=0.002), phylum Verrucomicrobia (OR [95% CI] = 1.166 [1.051 - 1.294], P=0.004), order Victivallales (OR [95% CI] = 1.268 [1.071 - 1.501], P=0.006), class Lentisphaeria (OR [95% CI] = 1.268 [1.071 - 1.501], P=0.006), and phylum Lentisphaerae (OR [95% CI] = 1.268 [1.071 - 1.501], P=0.006). In contrast, a negative correlation was found with the genus Candidatus Soleaferrea (OR [95% CI] = 0.817 [0.705 - 0.946], P=0.007). For robustness, we employed additional methods including MR Egger, Weighted Median, Simple Mode, and Weighted Mode, as detailed in **Table 3**.

**Table 3:** MR analysis of trauma exposure in MDD on the gut microbiota.

Classification	Methods	Nsnp	Beta	SE	OR(95%CI)	P value	Horizontal pleiotropy			Heterogeneity		MR-PRESSO	
							Egger intercept	SE	P value	Cochran’s Q	P value	outlier	P value
family Verrucomicrobiaceae	IVW	7	0.174	0.057	1.191 (1.066 - 1.33)	0.002	-0.346	0.172	0.101	2.504	0.776	0	0.387
	MR Egger		2.937	1.375	18.861 (1.274 - 279.157)	0.086							
	Weighted median		0.2	0.075	1.221 (1.054 - 1.415)	0.008							
	Simple mode		0.223	0.124	1.25 (0.981 - 1.594)	0.122							
	Weighted mode		0.24	0.122	1.271 (1.002 - 1.613)	0.096							
order Verrucomicrobiales	IVW	7	0.174	0.056	1.19 (1.065 - 1.329)	0.002	-0.346	0.172	0.101	2.496	0.777	0	0.43
	MR Egger		2.937	1.375	18.856 (1.274 - 279.086)	0.086							
	Weighted median		0.199	0.076	1.221 (1.052 - 1.416)	0.009							
	Simple mode		0.223	0.129	1.25 (0.971 - 1.61)	0.135							
	Weighted mode		0.24	0.115	1.271 (1.014 - 1.593)	0.083							
class Verrucomicrobiae	IVW	7	0.174	0.056	1.19 (1.065 - 1.329)	0.002	-0.346	0.172	0.101	2.496	0.777	0	0.412
	MR Egger		2.937	1.375	18.856 (1.274 - 279.086)	0.086							
	Weighted median		0.199	0.076	1.221 (1.051 - 1.417)	0.009							
	Simple mode		0.223	0.125	1.25 (0.979 - 1.596)	0.124							
	Weighted mode		0.24	0.115	1.271 (1.015 - 1.591)	0.081							
genus Akkermansia	IVW	7	0.174	0.057	1.19 (1.065 - 1.33)	0.002	-0.346	0.172	0.1	2.511	0.775	0	0.411
	MR Egger		2.939	1.375	18.895 (1.276 - 279.694)	0.086							
	Weighted median		0.201	0.076	1.222 (1.054 - 1.418)	0.008							
	Simple mode		0.226	0.123	1.254 (0.986 - 1.594)	0.115							
	Weighted mode		0.241	0.126	1.272 (0.994 - 1.628)	0.104							
**phylum Verrucomicrobia**	IVW	7	0.154	0.053	1.166 (1.051 - 1.294)	0.004	-0.322	0.168	0.115	2.138	0.83	0	0.496
	MR Egger		2.721	1.346	15.196 (1.086 - 212.602)	0.099							
	Weighted median		0.155	0.078	1.168 (1.003 - 1.36)	0.045							
	Simple mode		0.097	0.114	1.102 (0.881 - 1.379)	0.428							
	Weighted mode		0.163	0.118	1.178 (0.934 - 1.485)	0.217							
order Victivallales	IVW	7	0.238	0.086	1.268 (1.071 - 1.501)	0.006	0.118	0.272	0.682	3.24	0.663	0	0.784
	MR Egger		-0.708	2.176	0.492 (0.007 - 35.017)	0.758							
	Weighted median		0.197	0.114	1.217 (0.974 - 1.521)	0.084							
	Simple mode		0.189	0.165	1.208 (0.875 - 1.669)	0.295							
	Weighted mode		0.17	0.145	1.185 (0.891 - 1.576)	0.287							
class Lentisphaeria	IVW	7	0.238	0.086	1.268 (1.071 - 1.501)	0.006	0.118	0.272	0.682	3.24	0.663	0	0.792
	MR Egger		-0.708	2.176	0.492 (0.007 - 35.017)	0.758							
	Weighted median		0.197	0.113	1.217 (0.975 - 1.52)	0.083							
	Simple mode		0.189	0.169	1.208 (0.867 - 1.683)	0.306							
	Weighted mode		0.17	0.139	1.185 (0.902 - 1.557)	0.269							
phylum Lentisphaerae	IVW	7	0.237	0.086	1.268 (1.071 - 1.501)	0.006	0.112	0.272	0.698	3.254	0.661	0	0.8
	MR Egger		-0.657	2.175	0.519 (0.007 - 36.803)	0.775							
	Weighted median		0.189	0.116	1.207 (0.961 - 1.517)	0.105							
	Simple mode		0.187	0.15	1.206 (0.899 - 1.619)	0.258							
	Weighted mode		0.17	0.137	1.185 (0.906 - 1.55)	0.261							
genus Candidatus Soleaferrea	IVW	7	-0.202	0.075	0.817 (0.705 - 0.946)	0.007	0.253	0.238	0.337	2.929	0.711	0	0.658
	MR Egger		-2.22	1.902	0.109 (0.003 - 4.516)	0.296							
	Weighted median		-0.193	0.107	0.825 (0.669 - 1.016)	0.071							
	Simple mode		-0.186	0.143	0.83 (0.627 - 1.099)	0.241							
	Weighted mode		-0.176	0.137	0.839 (0.641 - 1.097)	0.247							

MR, Mendelian randomization; MDD, major depression disorder; Nsnp, Numbers of single nucleotide polymorphism; SE, Standard error; OR, Odds ratio; CI, Confidence interval; IVW, Inverse variance weighted.

MR-Egger regression and Cochran’s Q statistic analyses verified the absence of significant horizontal pleiotropy or heterogeneity among the IVs. Additionally, the MR-PRESSO test identified no significant deviations or outliers, thus strengthening the trustworthiness of our findings (**Table 3**). To further substantiate the robustness of our results related to Lentisphaerae, we conducted funnel plot and leave-one-out sensitivity analyses. These analyses affirmed the consistency and reliability of our causal estimates, as illustrated in **Supplementary Figures**.

## 4 Discussion

The connection between MDD and microbiome impacts was demonstrated by changes in the hypothalamic-pituitary-adrenal (HPA) axis and brain monoamine levels (25, 26). Early-life trauma and stress predispose individuals to high chances of MDD prevalence (4, 27). The knowledge of MDD pathogenesis after trauma is of fundamental importance for the development of effective prevention and treatment approaches. The metabolites originating from microbiota, such as short-chain fatty acids (SCFAs) and neurotransmitters, impact the microbiome-gut-brain axis, connecting neurocognitive functions to gut functions and contribute to neuropsychiatric disorder development (28–30). This axis is essential in understanding the onset of numerous neuropsychiatric disorders, emphasizing the central position that gut microbiota plays in neurobiological health (13, 26, 31–34). Newer studies indicated that the gut microbiota undergoes dysbiosis post-trauma that may result in the changes in the functioning of the brain and eventually give rise to posttraumatic neuropsychiatric disorders such as PTMDD (8, 14, 35–37). Traditional research methods, such as animal models and observational studies, have their limitations in providing conclusive evidence (8, 28). Therefore, linking the gut microbiota and PTMDD causally is still a difficult task.

In our study, we found that the phylum Verrucomicrobia is associated with a decreased risk of PTMDD. Additionally, reverse MR analysis revealed a positive association of PTMDD with specific taxa, including the genus Anaerotruncus, genus Butyrivibrio, phylum Lentisphaerae, and family Rhodospirillaceae. Conversely, a negative association was observed with the family Bacteroidales S24**–**7 group and genus Howardella, suggesting that PTMDD could influence the composition of the gut microbiota. Specific bacterial taxa increase the risk of developing PTMDD, and PTMDD, in turn, can alter the proportions of the gut microbiota, indicating complex crosstalk within the microbiome-gut-brain axis. Unlike the causal link we found between PTMDD and the gut microbiota, we did not observe any significant causal effects of the gut microbiota on trauma exposure in individuals with MDD. This suggests that the gut microbiota may exert causal effects only on specific subtypes of MDD that are directly triggered by trauma, rather than broadly influencing all trauma-related psychiatric outcomes. However, we discovered that trauma exposure in MDD has a positive impact on certain bacterial taxa, such as the family Verrucomicrobiaceae, order Verrucomicrobiales, class Verrucomicrobiae, genus Akkermansia, phylum Verrucomicrobia, order Victivallales, class Lentisphaeria, and phylum Lentisphaerae. In contrast, a negative impact was noted with the genus Candidatus Soleaferrea, indicating that trauma exposure in MDD could also alter the proportions of the gut microbiota. Importantly, the correlation of the phylum Verrucomicrobia with both PTMDD and trauma exposure in MDD underscores its potential role in neuropsychiatric responses to trauma, potentially affecting emotional and cognitive outcomes. This underscores the critical importance of the microbiome-gut-brain axis in psychiatric research, suggesting that targeting the gut microbiota could help manage or prevent trauma-related depressive symptoms. The number of chosen IVs for analyzing the effect of trauma exposure in MDD on the gut microbiota is fewer than 10. This poses a risk of weak instrument bias, which we addressed using MR-Egger regression, F-statistic testing, and by carefully interpreting the findings within the limitations of our study. To our knowledge, this is the first study to employ a bidirectional two-sample MR analysis to explore the gut microbiota’s role in PTMDD, and shedding new light on its nuanced relationship with PTMDD and opening avenues for novel therapeutic strategies.

The phylum Verrucomicrobia has been reported to play a vital role in human health. A study showed that levels of the phylum Verrucomicrobia are decreased in patients with progeria, and that dramatic increases in Verrucomicrobia can enhance both lifespan and health span in mice models (38).A large number of studies have shown that an increase in Akkermansia muciniphila, a member of Verrucomicrobia, can reduce the risk of the onset of several diseases, including diabetes, liver steatosis, obesity, cancers, and inflammation (39).Recent studies have demonstrated that Verrucomicrobia is also correlated with a variety of neuropsychiatric disorders and plays a role in the microbiome-gut-brain axis (40, 41).Verrucomicrobia play important roles in maintaining gut barrier integrity and modulating immune responses, which are connected with mood regulation (42). Compared to the controls, a decreased abundance of the phylum Verrucomicrobia was observed in children with autism spectrum disorder (43).Hemmings SMJ et al (36). found that a decrease in the phylum Verrucomicrobia is associated with the deterioration of symptoms in patients with PTSD. Akkermansia muciniphila, a specific species that belongs to the phylum Verrucomicrobia, has been reported to be involved in neuropsychiatric disorders, including depression, anxiety, and autism spectrum disorders (44). Some preclinical experiments have demonstrated that Akkermansia muciniphila can ameliorate depressive-like behavior in mice (45, 46).In our study, we found that the phylum Verrucomicrobia is associated with a decreased risk of developing PTMDD. However, it does not confer a similar benefit for individuals with MDD. This distinction suggests that Verrucomicrobia may specifically mitigate the risk of depression directly triggered by trauma, rather than generally lowering the predisposition to depression. Consequently, Verrucomicrobia holds potential as a targeted biomarker or therapeutic agent for a specific subtype of PTMDD. Additionally, our findings suggest that an increase in Verrucomicrobia among individuals with MDD following trauma exposure may represent an adaptive biological response, potentially aiding in coping mechanisms against post-traumatic psychological stress. These findings unveil the significant role of Verrucomicrobia in modulating the effects of trauma, opening new avenues for predictive diagnostics and targeted therapeutic strategies.

We found that both PTMDD and trauma exposure in individuals with MDD impacts the composition of the gut microbiota, suggesting the profound biological impacts of trauma. These changes in gut microbiota highlight the extensive influence that psychological stress and trauma can have on the body’s systems, demonstrating a crucial aspect of the bi-directional relationship between physical health and mental health conditions. On one hand, trauma can disrupt the balance of the gut microbiota, potentially leading to the development of neuropsychiatric sequelae, which is consistent with published literature (6, 8).On the other hand, there is an increase in certain beneficial bacteria, which helps the host adapt to these changes. For instance, higher levels of Verrucomicrobia and Lentisphaerae are associated with better sleep quality (47),which is known to influence the onset and severity of depression (48).Several studies have shown that a sustainable abundance of Akkermansia and Butyricimonas can alleviate depressive symptoms (46, 49).Bacillus licheniformis and Lactobacillales, recognized as probiotics, have been applied in pre-clinical trials to moderate anxiety and depression (50, 51).These bacteria can alleviate stress and reduce symptoms of MDD in the host by producing or influencing certain metabolites, such as SCFAs. SCFAs are recognized for their broad range of health benefits in mammals, including anti-inflammatory activity and potential neuroprotection (41).Such SCFAs can cross the blood-brain barrier and modulate neuroinflammation, neurotransmission, and brain cell integrity (52, 53). In addition, these metabolic processes are vital to energy production and might also contribute to energy homeostasis, altering brain function indirectly (54). Our findings reinforce the concept of the microbiome-gut-brain axis, illustrating how psychological states can not only alter the composition of the gut microbiota, potentially inducing mood disorders, but also how an increase in beneficial bacteria can facilitate mood regulation in response. These findings highlight the complex and reciprocal relationships within the gut-brain interaction network.

Our MR analysis boasts several strengths, firstly our use of a bidirectional MR approach to explore the complex interactions within the microbiome-gut-brain axis in PTMDD. This bi-directional communication system, linking the brain and gastrointestinal tract via neural, endocrine, and immune pathways, suggests that alterations in the gut microbiota following trauma exposure can significantly affect brain function and emotional states. Conversely, changes in neurological and emotional health can also alter the gut microbiota. Moreover, our findings reveal that trauma exposure in individuals with MDD impacts the gut microbiota composition, which can partly help alleviate symptoms of MDD. These observations further substantiate the critical role of the microbiome-gut-brain axis in MDD, particularly in trauma-exposed cases, underscoring its potential as a target for therapeutic intervention. Secondly, Both PTMDD and MDD with trauma exposure can alter the gut microbiota’s composition and diversity through lifestyle manipulations, such as diet, sleep-wake cycles, and response to stress. Inflammatory disorders that accompany PTMDD or MDD with trauma exposure could also influence the gut microbiota. This complicated interplay emphasizes the critical need for an integrated approach in treating these conditions, considering both mental and gastrointestinal health as interconnected systems within the broader framework of the microbiome-gut-brain axis. Thirdly, we found that the phylum Verrucomicrobia is correlated with both PTMDD and trauma exposure in MDD, suggesting that this bacterial group may play a significant role in the neuropsychiatric responses to trauma. This correlation indicates a potential influence of Verrucomicrobia on the emotional and cognitive outcomes following traumatic experiences. It highlights the importance of the gut-brain axis in psychiatric research and suggests that interventions targeting the gut microbiota could be beneficial in managing or even preventing the onset of trauma-related depressive symptoms. Lastly, alterations of gut microbiota following trauma create new perspectives for PTMDD therapy and prophylaxis. Compared to the genetic predispositions, social determinants and factors of aging which are mainly non-modifiable, gut microbiota presents with unique intervention avenues in PTMDD prevention and management. The gut microbiota manipulation, be it via administration of probiotics or alterations in diet, can resort to therapeutic measures for PTMDD symptomatology and hence showcases the potential of the gut microbiota as an overall pathophysiological approach to deal with trauma-associated psychiatric disorder.

This study provides insightful findings but is subject to certain limitations that must be carefully considered. Primarily, our focus on a European sample limits the generalizability of our results across different racial and ethnic groups, potentially overlooking crucial genetic and environmental variations that differ by population. Additionally, our reliance on GWAS data restricts our ability to account for the specific nature or timing of trauma, significant factors that can greatly influence the microbiome-gut-brain axis and its role in PTMDD. A further limitation arises from the use of retrospective self-reports for trauma exposure within the GWAS dataset, which may introduce bias. Such reports are susceptible to the respondents’ current mental state and personal traits, potentially skewing the accuracy of trauma accounts. Collectively, these limitations suggest that while our findings contribute meaningfully to the field, they should be interpreted with caution. Future research would benefit from more inclusive studies that involve diverse populations and utilize more precise data collection methods, such as medical records, to enhance the validity and generalizability of the results.

## 5 Conclusion

In conclusion, our bidirectional MR analysis robustly establishes a causal link between the gut microbiota and PTMDD, marking a significant advancement in the ongoing research into the microbiome-gut-brain axis. This finding highlights the axis’s crucial role in the development of PTMDD. Additionally, this study enhances our understanding of the complex gut-brain interactions and paves the way for further research into the treatment of post-traumatic neuropsychiatric disorders.

## Data availability statement

The datasets presented in this study can be found in online repositories. The names of the repository/repositories and accession number(s) can be found in the article/**Supplementary Material**.

## Ethics statement

Ethical approval was not required for the study involving humans in accordance with the local legislation and institutional requirements. Written informed consent to participate in this study was not required from the participants or the participants’ legal guardians/next of kin in accordance with the national legislation and the institutional requirements.

## Author contributions

SL: Writing – original draft, Writing – review & editing. WY: Investigation, Methodology, Writing – review & editing. YZ: Investigation, Writing – review & editing. WL: Funding acquisition, Project administration, Resources, Writing – review & editing. LZ: Writing – review & editing, Supervision, Conceptualization. LL: Writing – review & editing, Supervision, Conceptualization. YL: Conceptualization, Supervision, Writing – review & editing.
