# Supplementary material for: The gut microbiota and post-traumatic major depression disorder: insights from bidirectional two-sample Mendelian randomization
Source: Front Psychiatry. 2024 May 14;15:1383664. doi: 10.3389/fpsyt.2024.1383664 (PMC11130430; doi:10.3389/fpsyt.2024.1383664)
Supplement: Supplementary file 1 [file DataSheet_1.pdf]

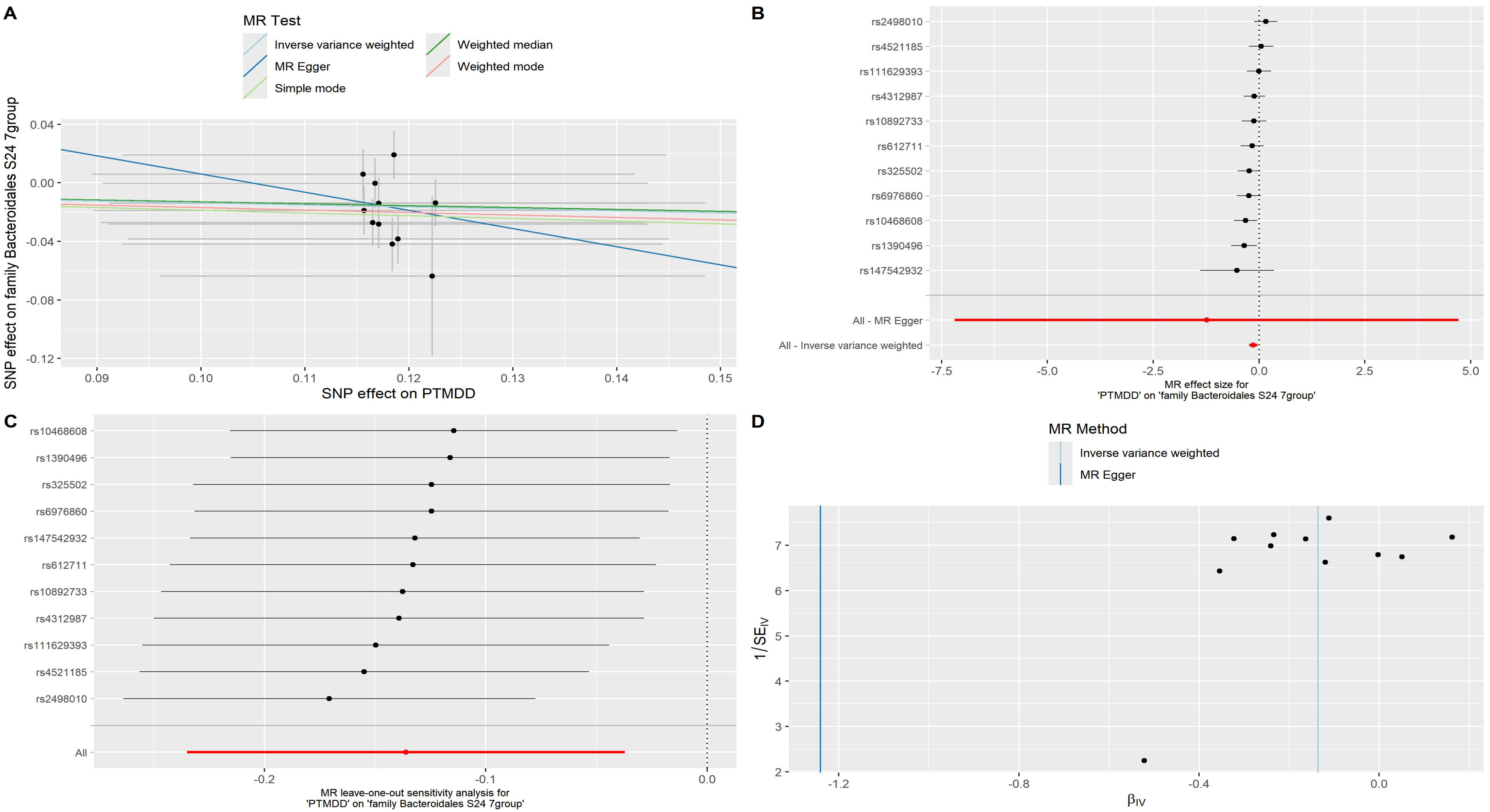

SUPPLEMENTARY FIGURE 1 . Forest plot (A), sensitivity analysis (B), scatter plot (C), and funnel plot (D) of the causal effect of the PTMDD on the family Bacteroidales S24 7group

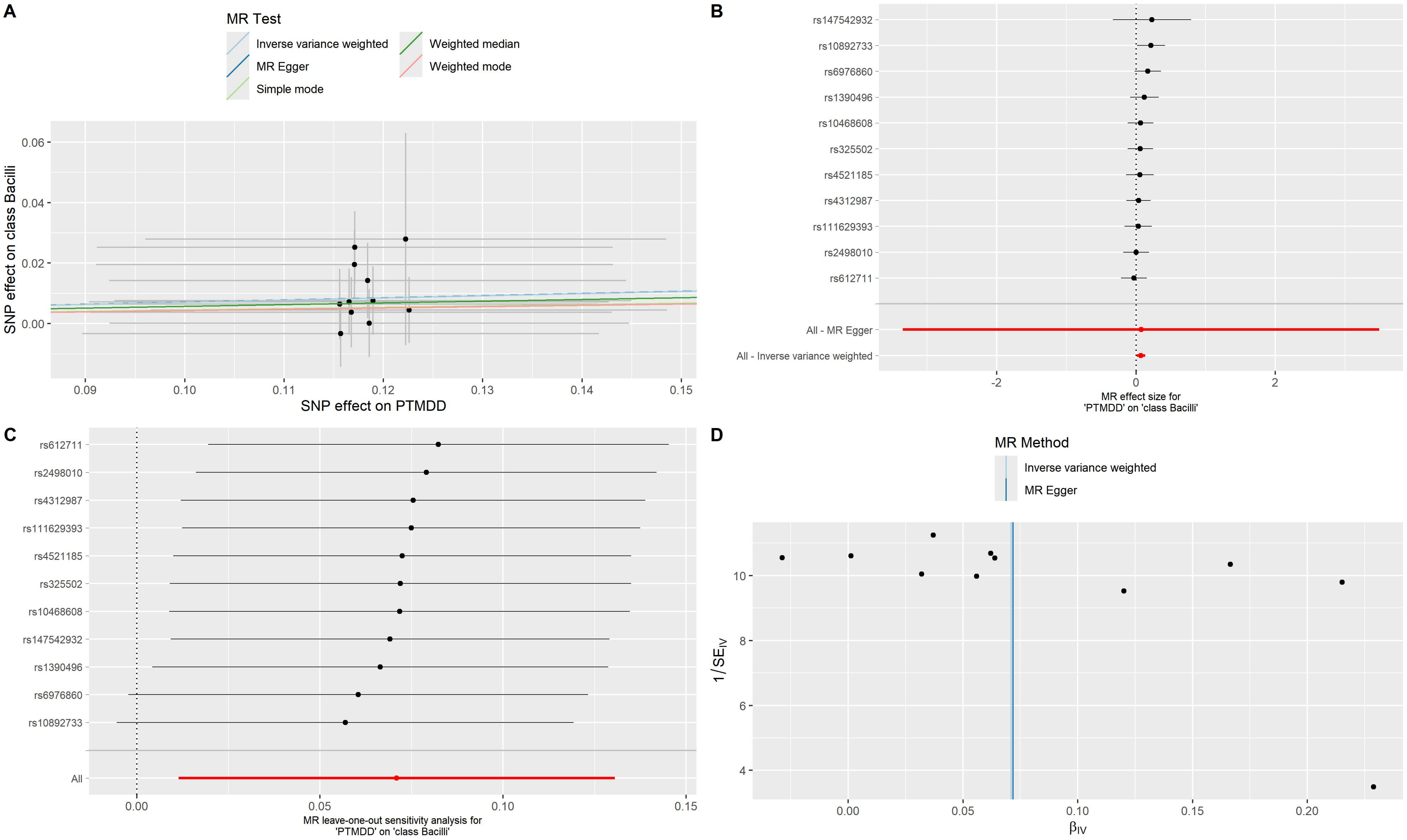

SUPPLEMENTARY FIGURE 2 . Forest plot (A), sensitivity analysis (B), scatter plot (C), and funnel plot (D) of the causal effect of the PTMDD on the class Bacilli

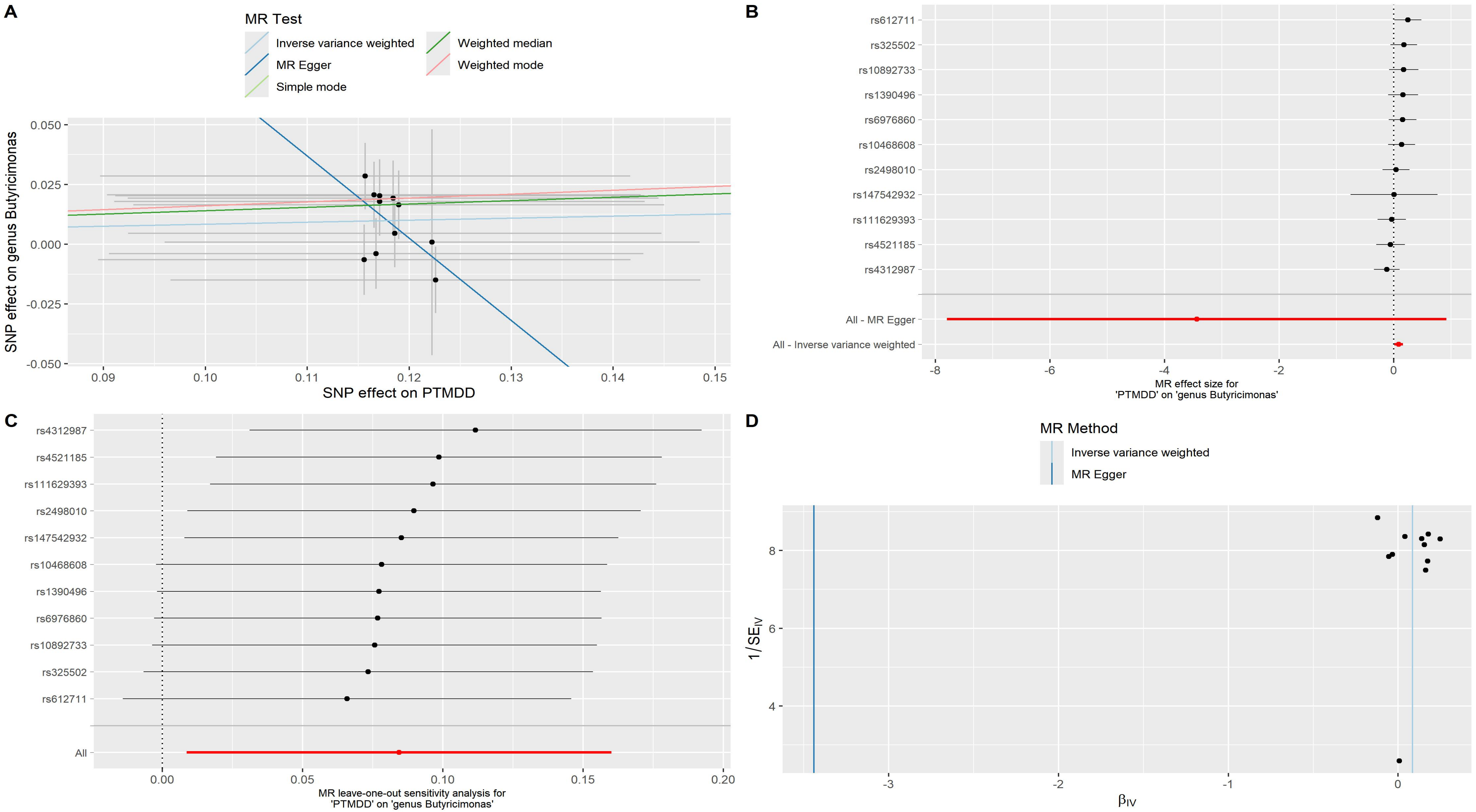

SUPPLEMENTARY FIGURE 3 . Forest plot (A), sensitivity analysis (B), scatter plot (C), and funnel plot (D) of the causal effect of the PTMDD on the genus Butyricimonas

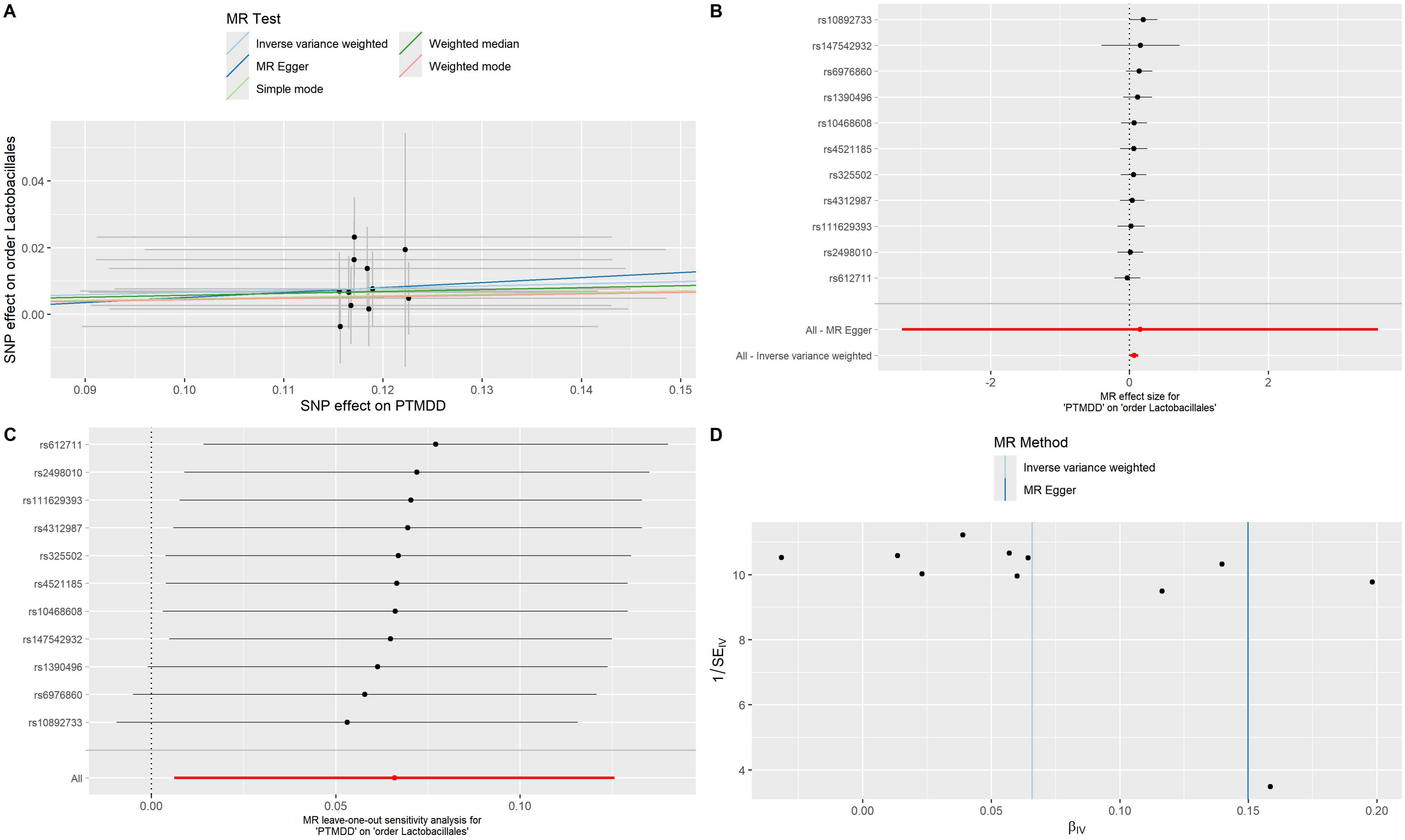

SUPPLEMENTARY FIGURE 4 . Forest plot (A), sensitivity analysis (B), scatter plot (C), and funnel plot (D) of the causal effect of the PTMDD on the order Lactobacillales

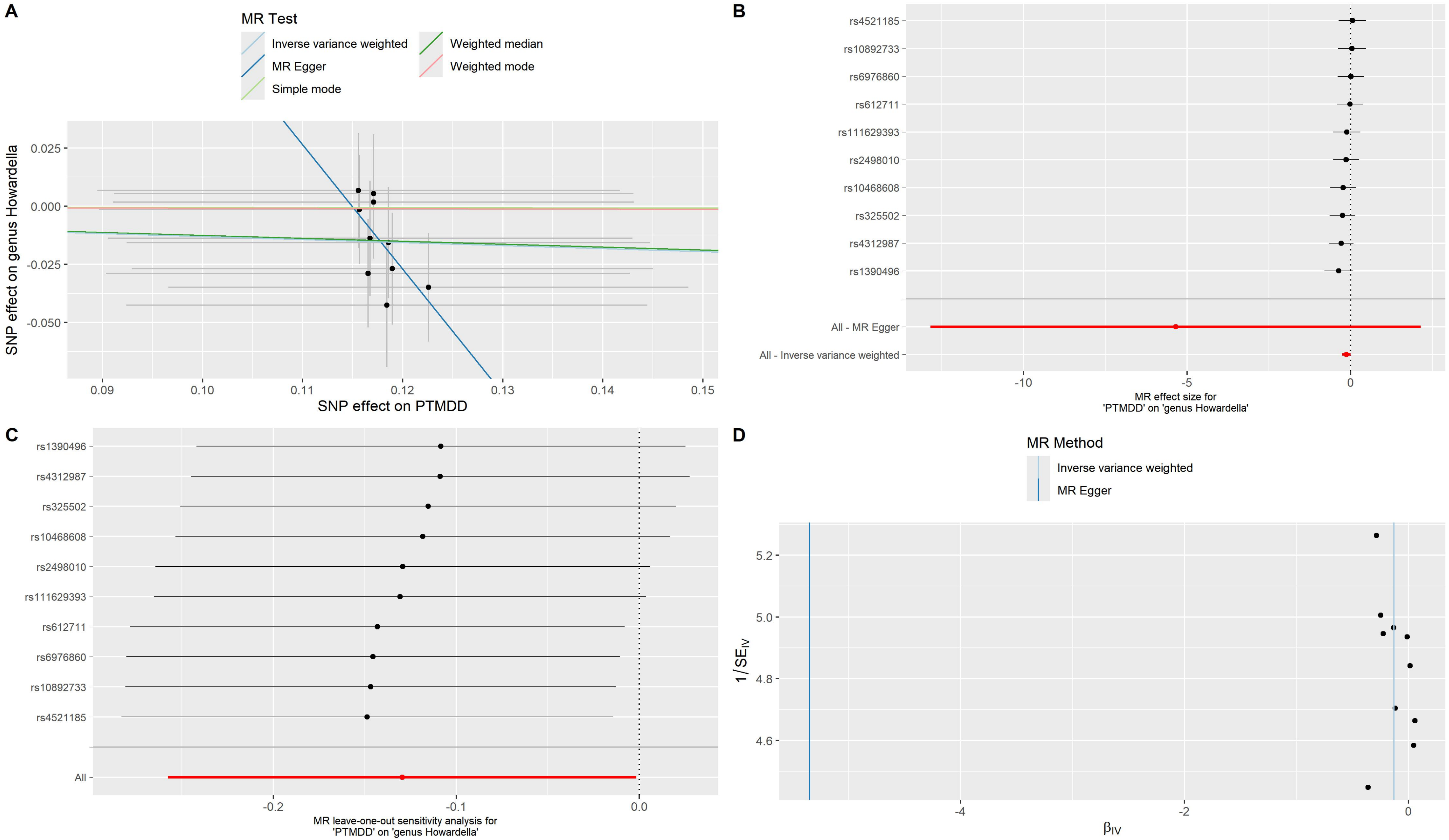

SUPPLEMENTARY FIGURE 5 . Forest plot (A), sensitivity analysis (B), scatter plot (C), and funnel plot (D) of the causal effect of the PTMDD on the genus Howardella

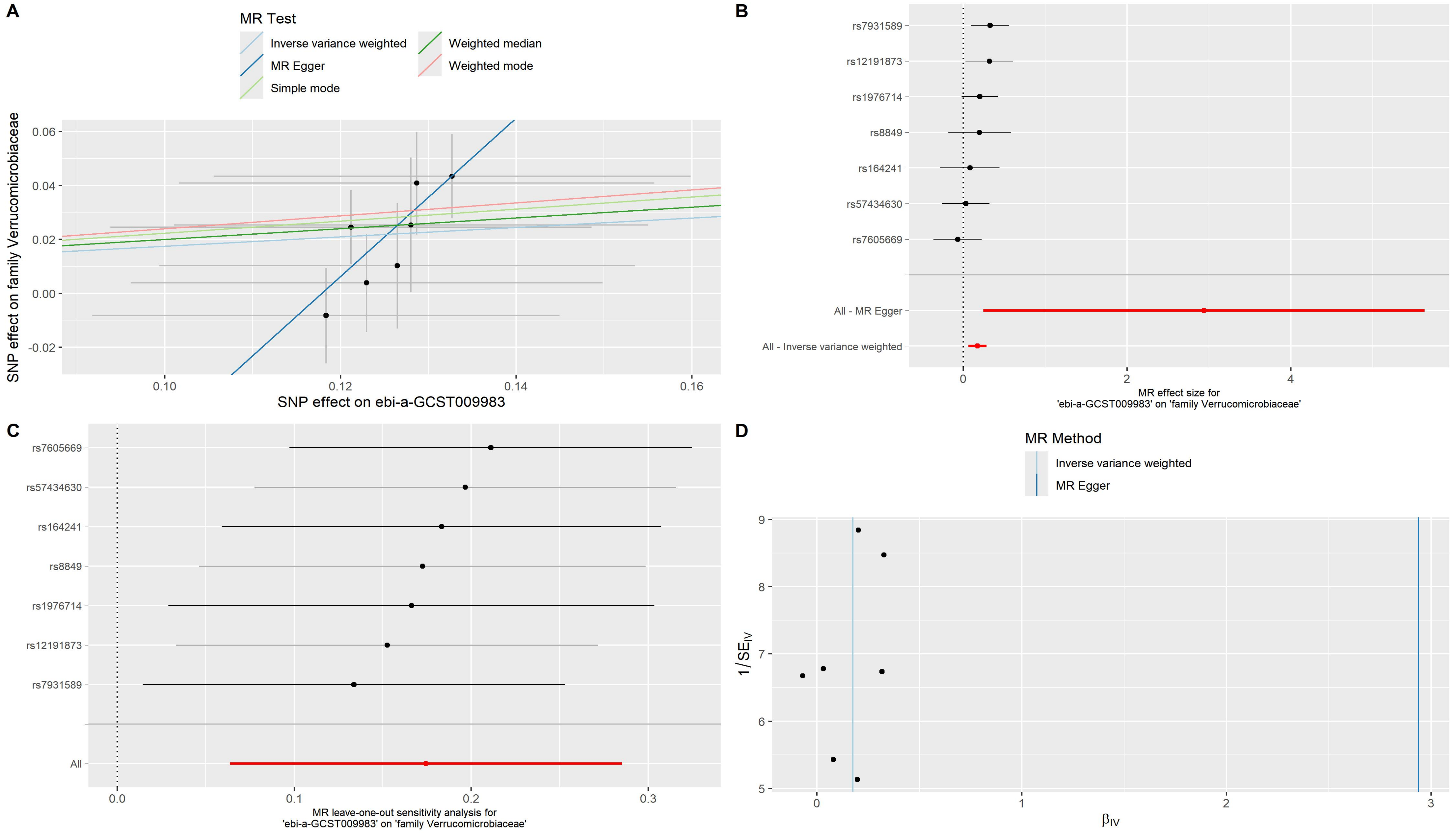

SUPPLEMENTARY FIGURE 6 . Forest plot (A), sensitivity analysis (B), scatter plot (C), and funnel plot (D) of the causal effect of the trauma exposure in MDD on the family Verrucomicrobiaceae

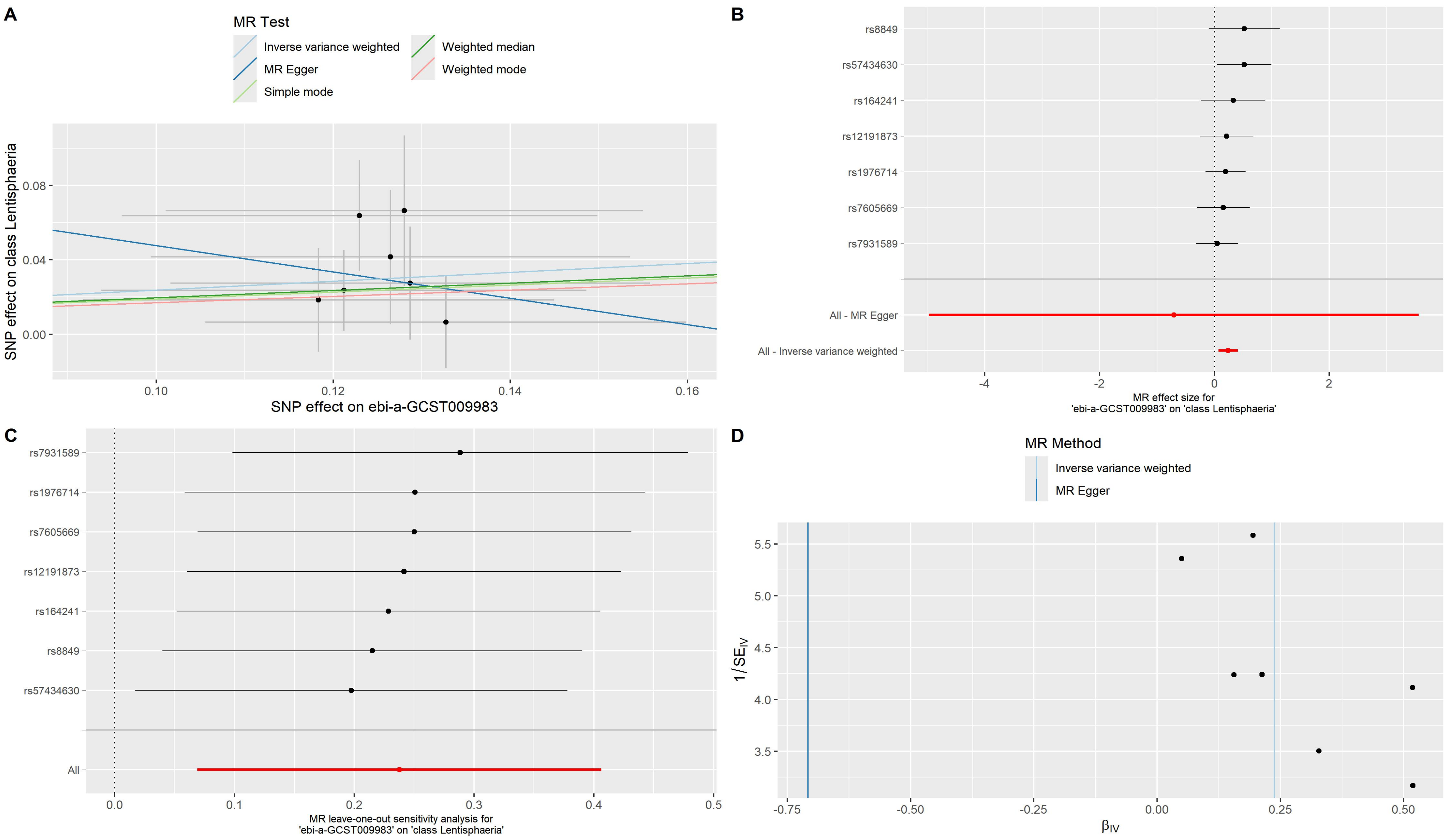

SUPPLEMENTARY FIGURE 7 . Forest plot (A), sensitivity analysis (B), scatter plot (C), and funnel plot (D) of the causal effect of the trauma exposure in MDD on the class Lentisphaeria

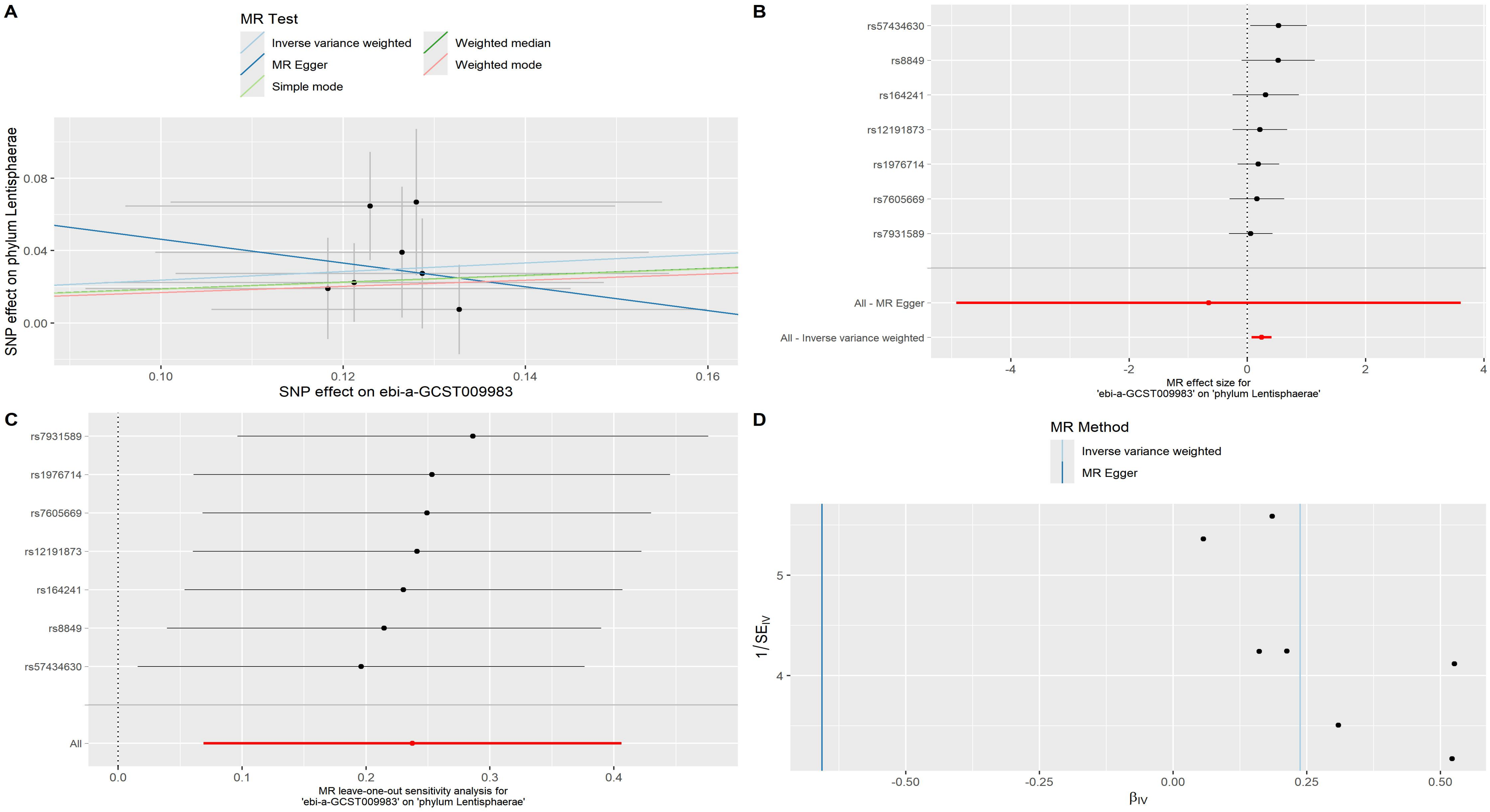

SUPPLEMENTARY FIGURE 8 . Forest plot (A), sensitivity analysis (B), scatter plot (C), and funnel plot (D) of the causal effect of the trauma exposure in MDD on the phylum Lentisphaerae

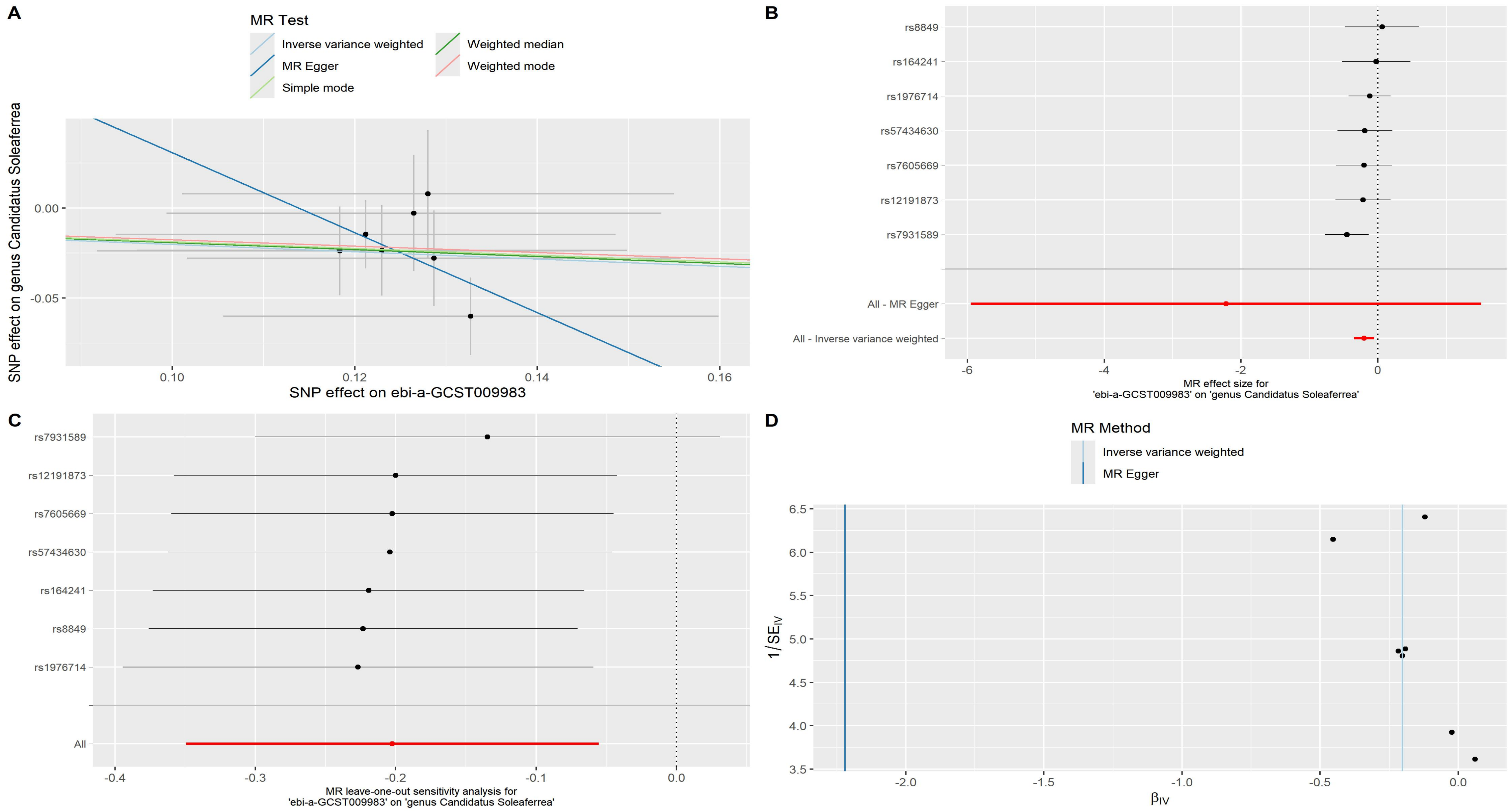

SUPPLEMENTARY FIGURE 9 . Forest plot (A), sensitivity analysis (B), scatter plot (C), and funnel plot (D) of the causal effect of the trauma exposure in MDD on the genus Candidatus Soleaferrea

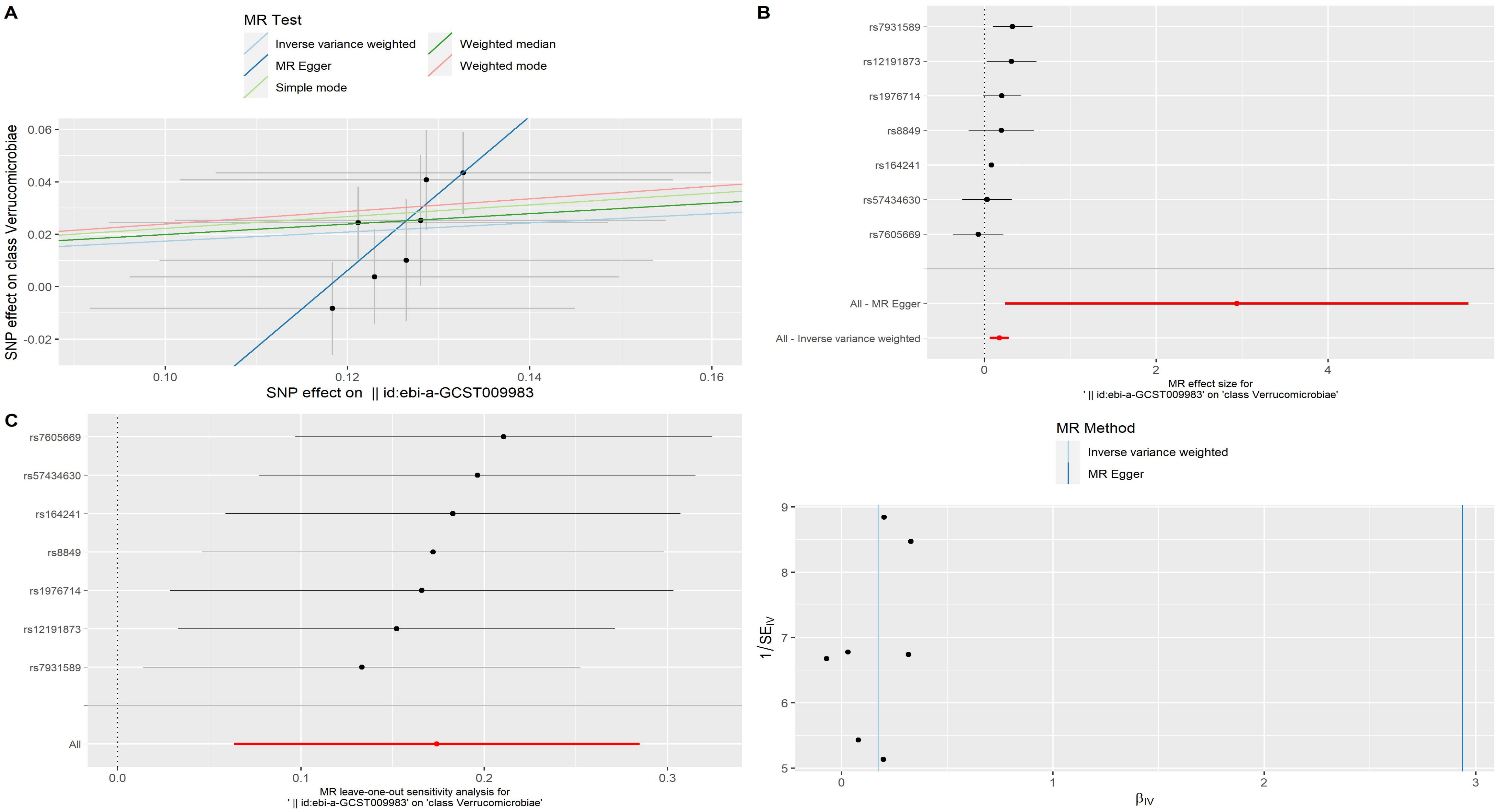

SUPPLEMENTARY FIGURE 10 . Forest plot (A), sensitivity analysis (B), scatter plot (C), and funnel plot (D) of the causal effect of the trauma exposure in MDD on the class Verrucomicrobiae

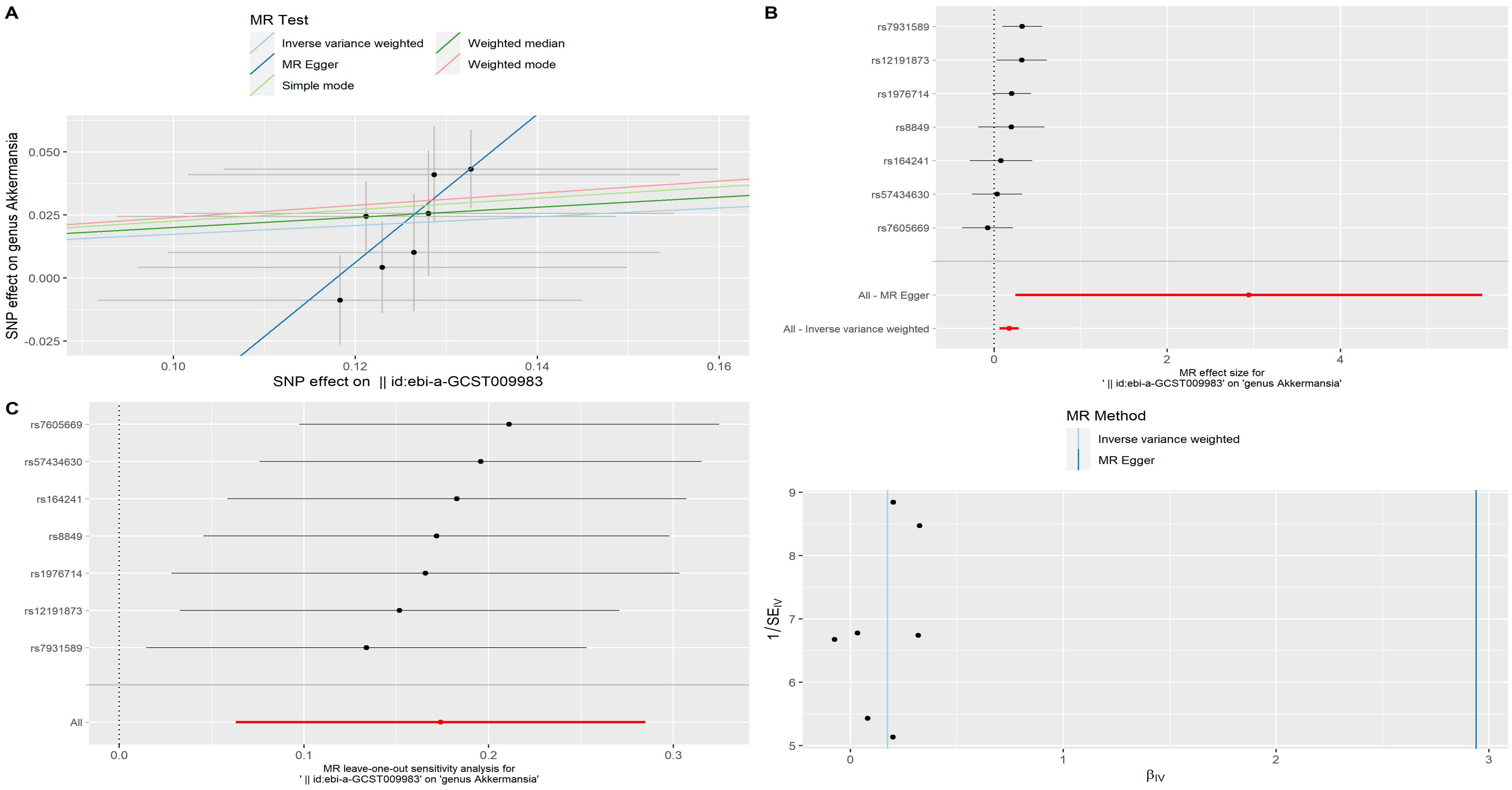

SUPPLEMENTARY FIGURE 11 . Forest plot (A), sensitivity analysis (B), scatter plot (C), and funnel plot (D) of the causal effect of the trauma exposure in MDD on the genus Akkermansia

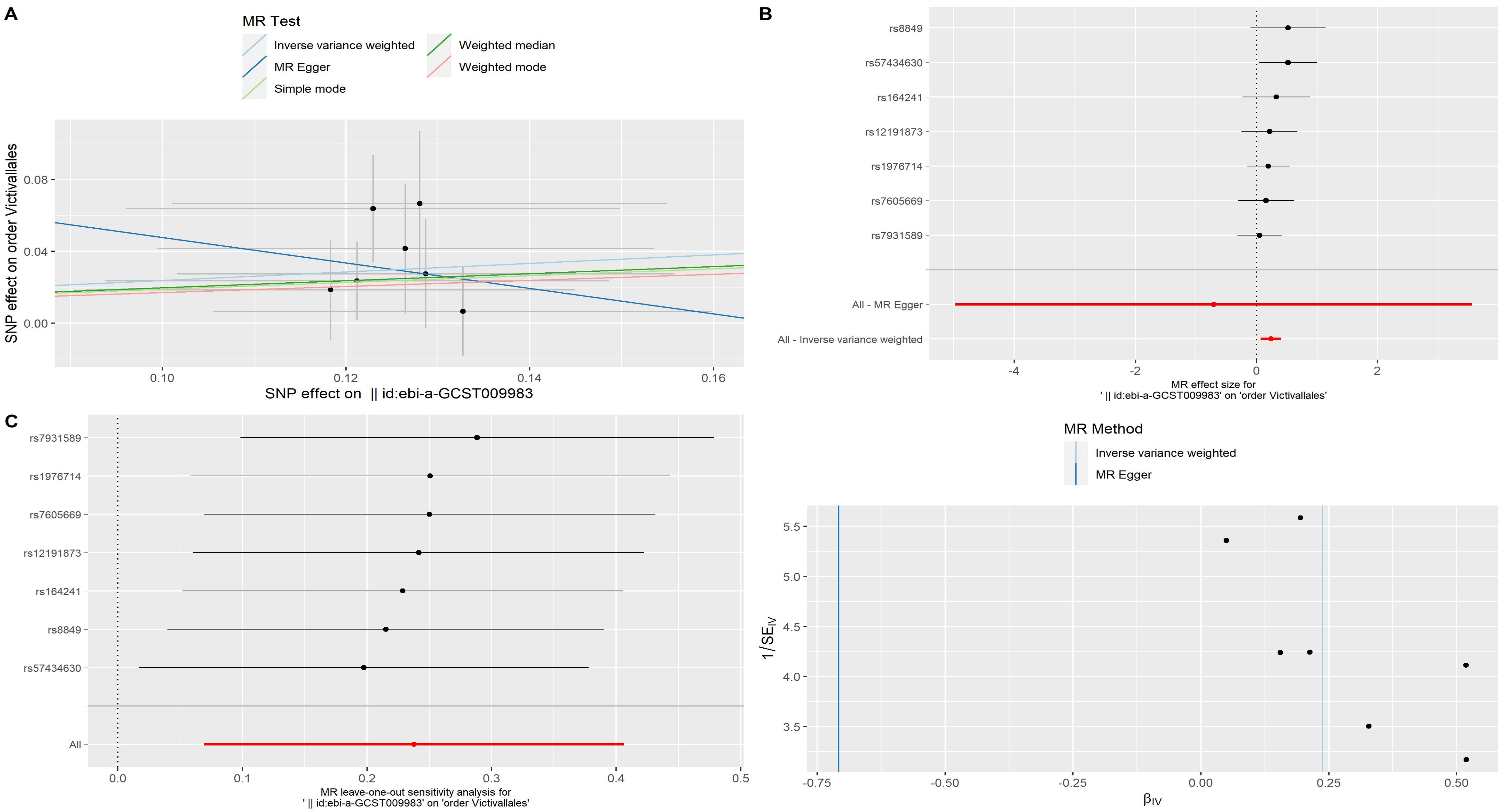

SUPPLEMENTARY FIGURE 12 . Forest plot (A), sensitivity analysis (B), scatter plot (C), and funnel plot (D) of the causal effect of the trauma exposure in MDD on the order Victivallales

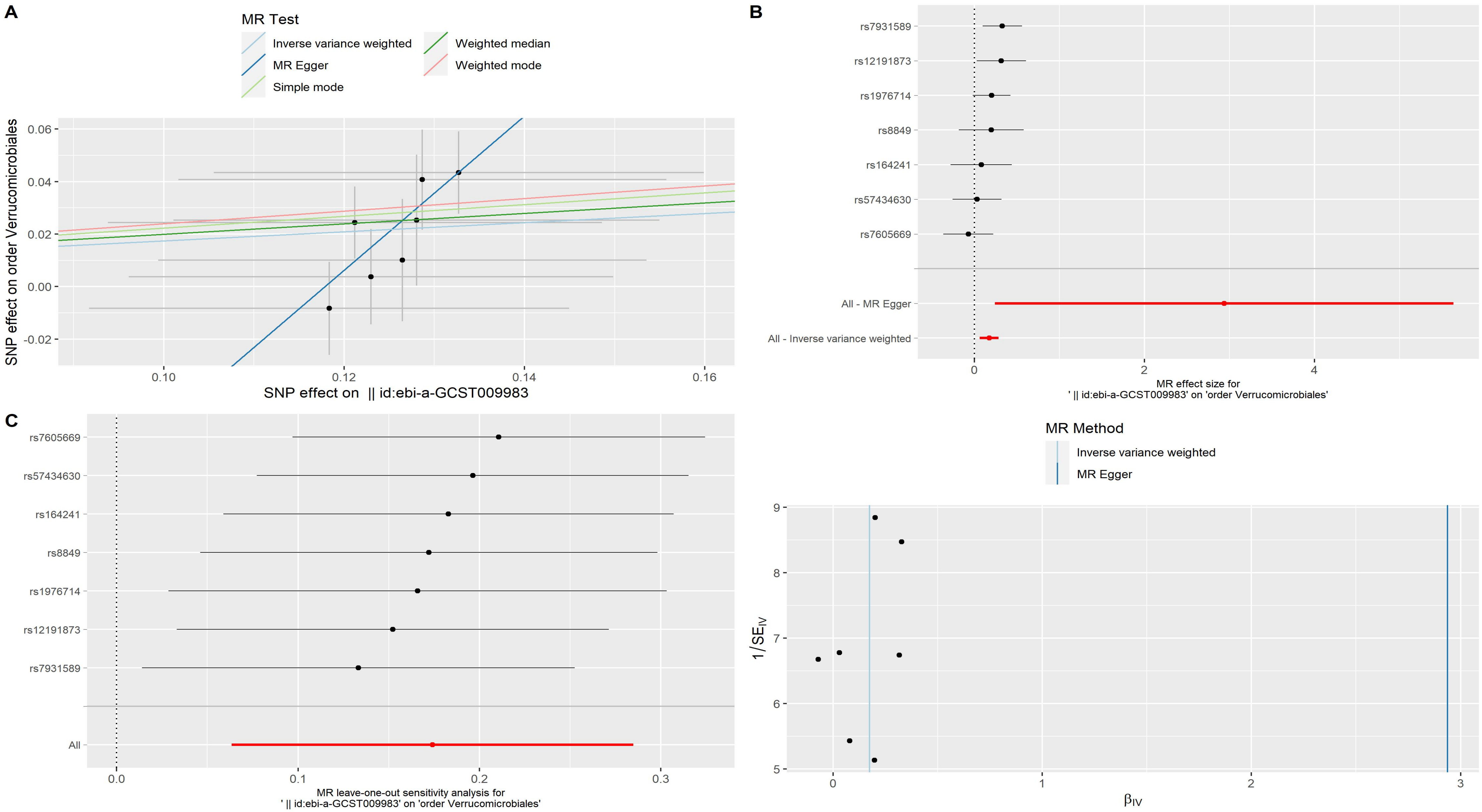

SUPPLEMENTARY FIGURE 13 . Forest plot (A), sensitivity analysis (B), scatter plot (C), and funnel plot (D) of the causal effect of the trauma exposure in MDD on the order Verrucomicrobiales

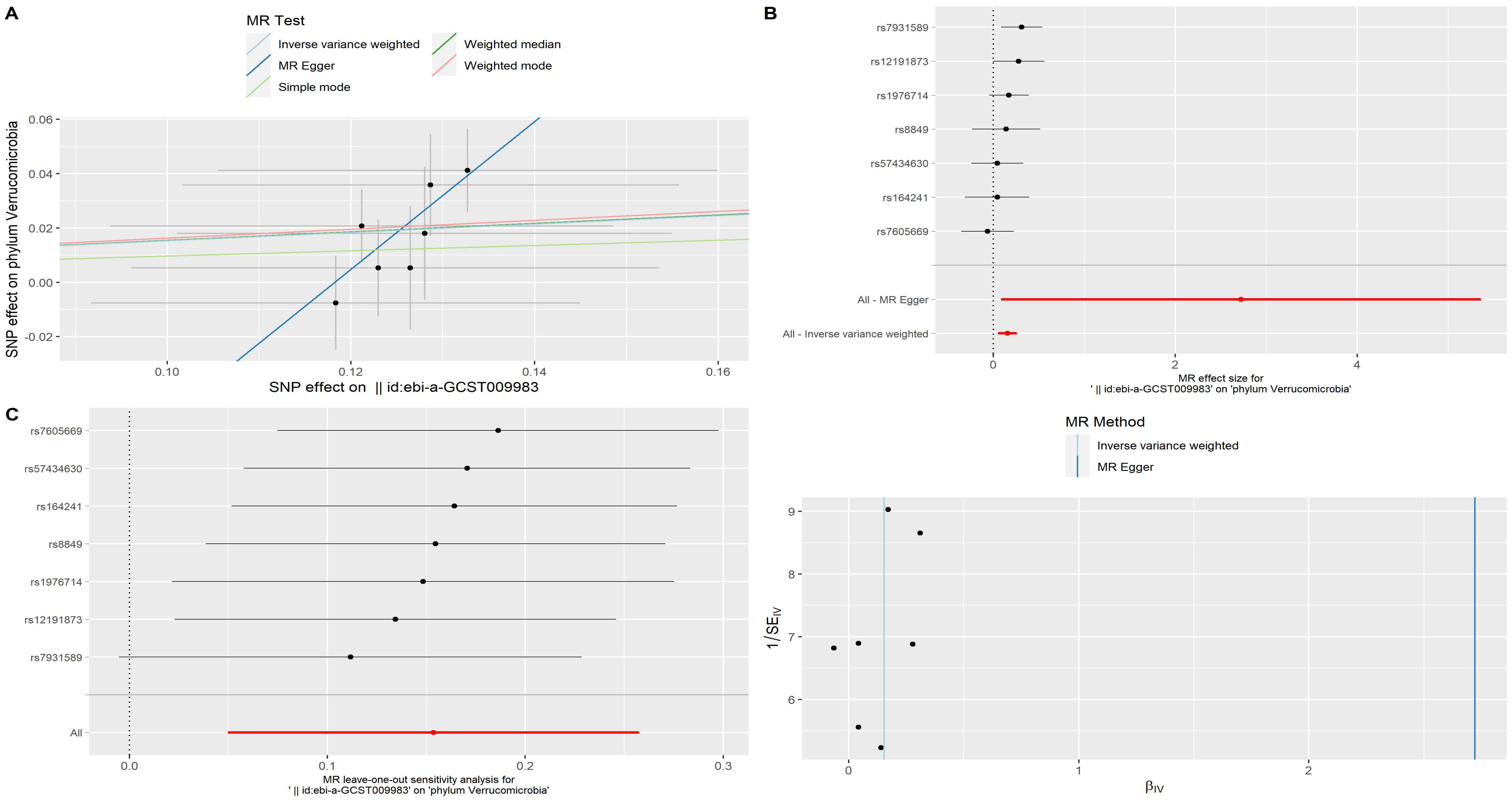

SUPPLEMENTARY FIGURE 14 . Forest plot (A), sensitivity analysis (B), scatter plot (C), and funnel plot (D) of the causal effect of the trauma exposure in MDD on the phylum Verrucomicrobia
