# Supplementary material for: The gut microbiota and post-traumatic major depression disorder: insights from bidirectional two-sample Mendelian randomization
Source: Front Psychiatry. 2024 May 14;15:1383664. doi: 10.3389/fpsyt.2024.1383664 (PMC11130430; doi:10.3389/fpsyt.2024.1383664)
Supplement: Supplementary file 3 [file Table_2.docx]

# FastMR

devtools::install_github("shaoming1995/fastMR")

Sys.setenv(GITHUB_TOKEN = "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")

install.packages("RSQLite", type = "binary")

remove.packages("RSQLite")

installed.packages()

devtools::install_local('C:\\Users\\Lenovo\\Desktop\\RSQLite')

devtools::install_github("r-dbi/RSQLite")

setwd("E:\\gut")

library(fastMR)

library(TwoSampleMR)

library(ggplot2)

library(plyr)

library(MendelianRandomization)

library(dplyr)

library(data.table)

expsplit_gut(name="DK2307006",

key=2994,

expfilename="exposure.csv")

Gut_IEU(

name="DK2307006",

key= ,

savefile="TMDDMR",

PATH="gut",

GWASID="ebi-a-GCST009980",

outname="PTMDD",

kb=10000,

r2=0.001)

IEU_Gut(

name="DK2307006",

key=2994,

savefile="MR OUTCOME",

PATH="E:\\gut",

GWASID="ebi-a-GCST009980",

expname="PTMMD",

p1=1e-05,

r2=0.001,

kb=10000)

PATH<-"E:\\gut2"

setwd(PATH)

library(ggplot2)

library(plyr)

library(MendelianRandomization)

library(TwoSampleMR)

EXP<-extract_instruments(outcomes = "ebi-a-GCST90017118", p1 = 1e-05,clump = T,r2=0.001,kb=10000, p2 = 1e-05,access_token = NULL)

EXP$id.exposure<-"phylum Verrucomicrobia"

EXP$exposure<-"phylum Verrucomicrobia"

or

EXP<-extract_instruments(outcomes = "ebi-a-GCST009983", p1 = 1e-05,clump = T,r2=0.001,kb=10000, p2 = 1e-05,access_token = NULL)

EXP$id.exposure<-"ebi-a-GCST009983"

EXP$exposure<-"ebi-a-GCST009983"

EXP$samplesize.exposure<-22880

head(EXP,6)

EXP$samplesize.exposure<-14306

OUT<-extract_outcome_data(snps=EXP$SNP,outcomes="ebi-a-GCST90016976",proxies=T,maf_threshold = 0.01,access_token = NULL)

OUT$outcome<-"genus Candidatus Soleaferrea"

OUT$id.outcome<-"genus Candidatus Soleaferrea"

OUT<-OUT[!duplicated(OUT$SNP),]

head(OUT)

EXP$samplesize.outcome<-14306

data_h<-harmonise_data(exposure_dat=EXP,outcome_dat=OUT,action=2)

data_h<-data_h%>%subset(data_h$mr_keep==TRUE)

head(data_h,6)

#write.csv(data_h,file="TRAUMA.csv")

SNP<-read.table("SNP.txt",header = T)

data_h_SNP<-merge(SNP,data_h,by="SNP",all=F)

data_h_SNP$samplesize.exposure<-22880

data_h_SNP_steiger<-steiger_filtering(data_h_SNP)

data_h_SNP_steiger<-data_h_SNP_steiger[!duplicated(data_h_SNP_steiger$SNP),]

mr<-mr(data_h_SNP_steiger)

mr_OR<-generate_odds_ratios(mr)

write.csv(mr_OR,file="mr_OR5.csv")

p1 <- mr_scatter_plot(mr[1:5,], data_h_SNP_steiger)

p1[[1]]

mr_outcome_loo <- mr_leaveoneout(data_h_SNP_steiger)

p3 <- mr_leaveoneout_plot(mr_outcome_loo)

p3[[1]]

mr_outcome_single <- mr_singlesnp(data_h_SNP_steiger)

p2 <- mr_forest_plot(mr_outcome_single)

p2[[1]]

mr_outcome_single <- mr_singlesnp(data_h_SNP_steiger)

p4 <- mr_funnel_plot(mr_outcome_single)

p4[[1]]

#install.packages("ggpubr")

library(ggpubr)

tiff(filename = "14.tiff", res = 300, width = 4500, height = 2700, compression ="lzw" )

ggarrange(p1[[1]],p2[[1]],p3[[1]],p4[[1]],labels=c("A","B","C","D"),nrow=2,ncol = 2)

dev.off()

H<-mr_heterogeneity(data_h_SNP_steiger)

H

ple <- mr_pleiotropy_test(data_h_SNP_steiger)

ple

data_s1<-data_h[,c("SNP","effect_allele.exposure","other_allele.exposure", "eaf.exposure",

"beta.exposure","se.exposure","pval.exposure","samplesize.exposure")]

data_s1$R2<-data_s1$beta.exposure*data_s1$beta.exposure*2*(data_s1$eaf.exposure)*(1-data_s1$eaf.exposure)

data_s1$Fvalue<-(data_s1$samplesize.exposure-2)*data_s1$R2/(1-data_s1$R2)

write.csv(data_s1,"3_R2_Ftrauma.csv",quote = F,row.names = F)

library(MRPRESSO)

mr_presso(BetaOutcome = "beta.outcome",BetaExposure="beta.exposure",

SdOutcome = "se.outcome", OUTLIERtest = T, DISTORTIONtest = T,SdExposure = "se.exposure",data=data_h_SNP_steiger)
